# Brazilian Ironstone Plant Communities as Reservoirs of Culturable Bacteria With Diverse Biotechnological Potential

**DOI:** 10.3389/fmicb.2018.01638

**Published:** 2018-07-23

**Authors:** Washington L. Caneschi, Érica B. Felestrino, Natasha P. Fonseca, Morghana M. Villa, Camila G. de C. Lemes, Isabella F. Cordeiro, Renata de A. B. Assis, Angélica B. Sanchez, Izadora T. Vieira, Luciana H. Y. Kamino, Flávio F. do Carmo, Camila C. M. Garcia, Leandro M. Moreira

**Affiliations:** ^1^Núcleo de Pesquisas em Ciências Biológicas, Universidade Federal de Ouro Preto, Ouro Preto, Brazil; ^2^Departamento de Ciências Biológicas, Instituto de Ciências Exatas e Biológicas, Universidade Federal de Ouro Preto, Ouro Preto, Brazil; ^3^Instituto Prístino, Belo Horizonte, Brazil

**Keywords:** canga, plasmids, PGPB, protection against ROS, arsenic tolerance

## Abstract

Extensive mineral extractivism in the Brazilian Iron Quadrangle (IQ) region has destroyed large areas of land, decimating plant species, and their associated microbiota. Very little is known about the microbiota of the region; hence, cultivable bacteria associated with plants of its soils were investigated for their biotechnological potential. Samples were collected from nine plant species and six soils, and 65 cultivable bacterial isolates were obtained. These represent predominantly gram-positive bacilli (70%) capable of producing amylases (55%), proteases (63%), cellulases (47%), indole acetic acid (IAA) (46%), siderophores (26%), and to solubilize phosphate (9%). In addition, 65% of these were resistant to ampicillin, 100% were sensitive to tetracycline, and 97% were tolerant to high arsenic concentrations. Three isolates were studied further: the isolate FOB3 (*Rosenbergiella* sp.) produced high concentrations of IAA *in vitro* in the absence of tryptophan – shown by the significant improvement in plant germination and growth rate where the isolate was present. For isolates C25 (*Acinetobacter* sp.) and FG3 (*Serratia* sp.), plasmids were purified and inserted into *Escherichia coli* cells where they modified the physiological profile of the transformed strains. The *E. coli*::pFG3B strain showed the highest capacity for biofilm production, as well as an increase in the replication rate, arsenic tolerance and catalase activity. Moreover, this strain increased DNA integrity in the presence of arsenic, compared to the wild-type strain. These results help to explain the importance of bacteria in maintaining plant survival in ferruginous, rocky soils, acting as plant growth promoters, and to highlight the biotechnological potential of these bacteria.

**IMPORTANCE**

The Iron Quadrangle region is responsible for ∼60% of all Brazilian iron production and, at the same time, is responsible for housing a wide diversity of landscapes, and consequently, a series of endemic plant species and dozens of rare species – all of which have been poorly studied. Studies exploring the microbiota associated with these plant species are limited and in the face of the continuous pressure of extractive action, some species along with their microbiota are being decimated. To understand the potential of this microbiota, we discovered that cultivable bacterial isolates obtained from plants in the ferruginous rocky soil of the Iron Quadrangle region have diverse biotechnological potential, revealing a genetic ancestry still unknown.

## Introduction

The ironstone plant communities are hotspots for biodiversity and endemism in metal-rich regions. Owing to their restricted distribution and association with the main deposits of iron ore in the world, these hotspots are among some of the most threatened vegetations (Gibson et al., 2010). In southeastern Brazil, these plant communities occur mainly in the Iron Quadrangle (IQ): a mineral province formed by old, geologically complex lands, distributed over an area of ∼ 7,200 km^2^. This region presents heterogeneity of reliefs, phytophysiognomies, and landscapes, intensely threatened by the high concentration of mineral complexes, which, in turn, generate technological and environmental challenges for the management of immense environmental liabilities, including the recovery of degraded areas (Dorr, 1969; Jacobi et al., 2011; Sonter et al., 2014; Carmo et al., 2017). These attributes have classified the IQ as an area of extremely high importance for the conservation and sustainable use of Brazilian biodiversity (MMA, 2007).

The ironstone outcrop plant communities are associated with iron duricrusts (known as “cangas”), formed from the intense weathering of banded iron formations (BIFs) over millions of years. These plant communities are exposed to conditions that determine severe restrictions for the establishment of plants, such as high ultraviolet (UV) exposure; extreme scarcity of soil, which makes fixing and root growth more difficult; high concentrations of metals, specifically Fe, Mn, and Al; acidity and very low nutrient availability (Jacobi et al., 2007; Schaefer et al., 2015; Carmo and Jacobi, 2016). Moreover, developed soils of metalliferous substrates can promote the selection of plant species resistant to high levels of metals, by means of physiological and morphological adaptations (Vincent and Meguro, 2008).

Plant adaptive mechanisms to abiotic stresses (e.g., salinity, heat, water scarcity, and metal concentration) are complex, involving the reception and translation of signals, followed by genetic and physiological responses. Among the most common adaptive mechanisms are the production of osmolytes, alterations in water transport and the elimination of reactive species (RS) (Rodriguez et al., 2008). However, this adaptation goes beyond genetic and physiological characteristics, depending also on the presence of endophytic or rhizosphere microorganisms, which play a fundamental role in this adaptive process (Souza et al., 2015).

The soil is characterized as an environment replete with microorganisms, including bacteria, fungi, actinomycetes, protozoa, viruses, and algae. Among these, bacteria are the most abundant, and can maintain both beneficial and harmful ecological relationships with plants. Bacteria that benefit plants are called plant growth promoting bacteria (PGPB). These benefits include direct involvement with mechanisms, such as the acquisition of mineral resources (iron, nitrogen, phosphate) or even by the production of molecules that mimic phytorhormones (Glick, 2012; Souza et al., 2015). In addition, PGPB can also indirectly benefit the plant community by reducing the harmful effects of phytopathogens or even by removing xenobiotics from numerous compounds harmful to plant growth (Glick, 2012; Kong and Glick, 2017). Knowledge of the microorganisms of these ancient ecosystems is still incipient.

From an ecological perspective, plants of the IQ are under imminent risk of decimation by anthropic actions, mainly mineral extraction (Jacobi et al., 2011). In this context, loss of flora would most likely induce the loss of specialized microbiota. This makes the IQ an excellent area of study for the prospection of neglected microorganisms.

In order to reduce this gap in biological knowledge, bacteria associated with nine ironstone plant species of the IQ were investigated. After cultivation, isolated bacteria were submitted to a series of biochemical and morphological characterization tests, that allowed the identification of the biotechnological potential in this unexplored genetic heritage.

## Materials and Methods

### Study Area and Sampling of Soil and Plants

**FIGURE 1:**
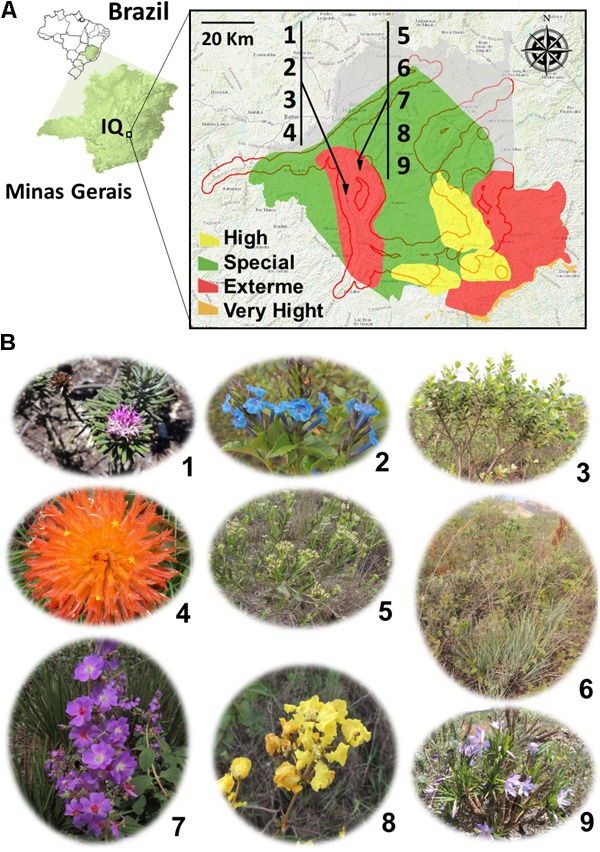
**(A)** Localization of collection areas for the morphological and biochemical characterization of bacterial isolates obtained from plant species and soils from the Iron Quadrangle (MG) region, southeastern Brazil. The numbers 1–9 indicate the plant species sampled and identified in **Table 1** and shown in **(B)**. The red contour determines the edges of the Iron Quadrangle while the colors yellow, red, orange, and green the degree of importance of the region for preservation of the flora of the Minas Gerais state.

In the Iron Quadrangle (IQ), southeastern Brazil (**Figure 1**), the BIFs of the Itabira geological group (2.6–2.1 Ga, paleoproterozoic) and associated cangas stand out (Rosière and Chemale, 2000). In this region, a subtropical climate predominates, which, according to Köppen climate classification, is characterized by dry winters and rainy summers. The average temperature of the coldest month is 18°C and the hottest month is 22°C. The samples were collected from cangas (iron duricrusts) located in Sinclinal Moeda, Nova Lima and Jardim Canada, Minas Gerais (latitude 20°15′83^′′^S, longitude 43°97′41^′′^W). Six samples of soil and nine species of plants were collected: *Lychnophora pinaster* Mart., *Stachytarpheta glabra* Cham., *Baccharis reticularia* DC., *Gomphrena arborescens* L.f., *Symphyopappus compressus* (Gardner) B. L. Rob., *Lagenocarpus rigidus* Nees, *Pleroma heteromallum* D. Don (D. Don), *Peixotoa tomentosa* A. Juss., and *Vellozia compacta* Mart. ex Schult. & Schult.f. (**Figure 1** and **Table 1**).

**Table 1:** Plants and soils sampled, and bacterial isolates obtained.

Plant samples				
	
ID	Scientific name	Canga location	Source	Isolate name
1	*Lychnophora pinaster* Mart.	Mãe d’agua (Sinclinal Moeda)	Flower	FA1
	*Lychnophora pinaster* Mart.	Mãe d’agua (Sinclinal Moeda)	Root	RA1
	*Lychnophora pinaster* Mart.	Mãe d’agua (Sinclinal Moeda)	Root	RA2
	*Lychnophora pinaster* Mart.	Mãe d’agua (Sinclinal Moeda)	Root	RA3
	*Lychnophora pinaster* Mart.	Mãe d’agua (Sinclinal Moeda)	Root	RA4
	*Lychnophora pinaster* Mart.	Mãe d’agua (Sinclinal Moeda)	Root	RA5
	*Lychnophora pinaster* Mart.	Mãe d’agua (Sinclinal Moeda)	Rhizosphere	RizA1
	*Lychnophora pinaster* Mart.	Mãe d’agua (Sinclinal Moeda)	Rhizosphere	RizA2
2	*Stachytarpheta glabra* Cham.	Mãe d’agua (Sinclinal Moeda)	Flower	FG1
	*Stachytarpheta glabra* Cham.	Mãe d’agua (Sinclinal Moeda)	Flower	FG2
	*Stachytarpheta glabra* Cham.	Mãe d’agua (Sinclinal Moeda)	Flower	FG3
3	*Baccharis reticularia* DC.	Mãe d’agua (Sinclinal Moeda)	Flower	FB1
	*Baccharis reticularia* DC.	Mãe d’agua (Sinclinal Moeda)	Flower	FB2
	*Baccharis reticularia* DC.	Mãe d’agua (Sinclinal Moeda)	Flower	FB3
	*Baccharis reticularia* DC.	Mãe d’agua (Sinclinal Moeda)	Flower	FB4
	*Baccharis reticularia* DC.	Mãe d’agua (Sinclinal Moeda)	Leaf	FoB1
	*Baccharis reticularia* DC.	Mãe d’agua (Sinclinal Moeda)	Leaf	FoB2
	*Baccharis reticularia* DC.	Mãe d’agua (Sinclinal Moeda)	Leaf	FoB3
4	*Gomphrena arborescens* L.f.	Mãe d’agua (Sinclinal Moeda)	Leaf	FPT1
	*Gomphrena arborescens* L.f.	Mãe d’agua (Sinclinal Moeda)	Flower	IPT1
	*Gomphrena arborescens* L.f.	Mãe d’agua (Sinclinal Moeda)	Flower	IPT2
	*Gomphrena arborescens* L.f.	Mãe d’agua (Sinclinal Moeda)	Flower	IPT3
	*Gomphrena arborescens* L.f.	Mãe d’agua (Sinclinal Moeda)	Root	RPT1
	*Gomphrena arborescens* L.f.	Mãe d’agua (Sinclinal Moeda)	Root	RPT2
	*Gomphrena arborescens* L.f.	Mãe d’agua (Sinclinal Moeda)	Root	RPT3
	*Gomphrena arborescens* L.f.	Mãe d’agua (Sinclinal Moeda)	Rhizosphere	RizPT1
	*Gomphrena arborescens* L.f.	Mãe d’agua (Sinclinal Moeda)	Rhizosphere	RizPT2
	*Gomphrena arborescens* L.f.	Mãe d’agua (Sinclinal Moeda)	Rhizosphere	RizPT3
	*Gomphrena arborescens* L.f.	Mãe d’agua (Sinclinal Moeda)	Tubercle	TPT1
	*Gomphrena arborescens* L.f.	Mãe d’agua (Sinclinal Moeda)	Tubercle	TPT2
	*Gomphrena arborescens* L.f.	Mãe d’agua (Sinclinal Moeda)	Tubercle	TPT3
	*Gomphrena arborescens* L.f.	Mãe d’agua (Sinclinal Moeda)	Tubercle	TPT4
	*Gomphrena arborescens* L.f.	Mãe d’agua (Sinclinal Moeda)	Tubercle	TPT5
	*Gomphrena arborescens* L.f.	Mãe d’agua (Sinclinal Moeda)	Tubercle	TPT6
5	*Symphyopappus compressus* (Gardner) B. L. Rob.	Jardim Canadá (Nova Lima)	Root	RS1
	*Symphyopappus compressus* (Gardner) B. L. Rob.	Jardim Canadá (Nova Lima)	Root	RS2
6	*Lagenocarpus rigidus* Nees	Jardim Canadá (Nova Lima)	Leaf	LR1
7	*Pleroma heteromallum* D. Don (D. Don)	Jardim Canadá (Nova Lima)	Leaf	TH1
8	*Peixotoa tomentosa* A. Juss.	Jardim Canadá (Nova Lima)	Flower	P1
	*Peixotoa tomentosa* A. Juss.	Jardim Canadá (Nova Lima)	Flower	P2
9	*Vellozia compacta* Mart. ex Schult. & Schult.f.	Jardim Canadá (Nova Lima)	Leaf	FCE
	*Vellozia compacta* Mart. ex Schult. & Schult.f.	Jardim Canadá (Nova Lima)	Root	RCE1
	*Vellozia compacta* Mart. ex Schult. & Schult.f.	Jardim Canadá (Nova Lima)	Root	RCE2
	*Vellozia compacta* Mart. ex Schult. & Schult.f.	Jardim Canadá (Nova Lima)	Root	RCE3
	*Vellozia compacta* Mart. ex Schult. & Schult.f.	Jardim Canadá (Nova Lima)	Root	RCE4
	
**Soil samples**				
	
1	Canga 1	Jardim Canadá (Nova Lima)	Soil	C11
	Canga 1	Jardim Canadá (Nova Lima)	Soil	C12
	Canga 1	Jardim Canadá (Nova Lima)	Soil	C13
2	Canga 2	Jardim Canadá (Nova Lima)	Soil	C21
	Canga 2	Jardim Canadá (Nova Lima)	Soil	C22
	Canga 2	Jardim Canadá (Nova Lima)	Soil	C23
	Canga 2	Jardim Canadá (Nova Lima)	Soil	C24
	Canga 2	Jardim Canadá (Nova Lima)	Soil	C25
3	Canga 3	Jardim Canadá (Nova Lima)	Soil	C31
	Canga 3	Jardim Canadá (Nova Lima)	Soil	C32
	Canga 3	Jardim Canadá (Nova Lima)	Soil	C33
4	Canga 4	Jardim Canadá (Nova Lima)	Soil	C41
	Canga 4	Jardim Canadá (Nova Lima)	Soil	C42
	Canga 4	Jardim Canadá (Nova Lima)	Soil	C43
	Canga 4	Jardim Canadá (Nova Lima)	Soil	C44
	Canga 4	Jardim Canadá (Nova Lima)	Soil	C45
5	Canga 5	Jardim Canadá (Nova Lima)	Soil	C51
6	Canga 6	Jardim Canadá (Nova Lima)	Soil	C61
	Canga 6	Jardim Canadá (Nova Lima)	Soil	C62
	Canga 6	Jardim Canadá (Nova Lima)	Soil	C63

Total of isolates				65


### Isolation and Preservation of Microorganisms

The plant samples (stems, roots, and leaves) were initially washed with distilled water. Then, they were immersed in 2.5% sodium hypochlorite solution for 2 min, then washed in 70% ethanol for 2 min. Finally, the samples were washed with sterile distilled water and placed on plates containing Luria Bertani (LB) agar, pH 7.0, containing 0.03 mg/l of antifungal methyl thiophanate. The samples were incubated at 28°C for 3–4 days for bacterial growth. Isolated colonies were collected with sterile toothpicks and grown on nutrient agar (NA). After isolation, the bacterial cultures grown in liquid LB were supplemented with 30% glycerol and stored at –80°C.

### Construction of a Matrix Plate for Biochemical Assays

For the large-scale assays, a 96 well array was constructed in which all isolates were grown in casein nutrient agar (CN) for 48 h at 28°C, then transferred using a multi-inoculator to Petri dishes or 96-well plates (according to the related biochemical assay).

### Indole Acetic Acid (IAA) Production

To analyze IAA production, the colorimetric method adapted from BRIC and collaborators was used (Bric et al., 1991). The isolates were grown in a U-shaped bottom 96-well plate (Costar^TM^) containing CN enriched with 5 mM L-tryptophan for 2 days at 28°C. The plates were centrifuged at 0.7 ×*g* and 100 μl of the supernatant was transferred to a new 96 well plate. Afterward, 100 μl of Salkowski solution (2 mL 0.5 M FeCl_3_.6H_2_O/l of HClO_4_) was added to each well and allowed to stand for 2 h. Subsequently, the color change from yellow to brown was verified, indicating hormone production. A calibration curve with different concentrations of IAA was constructed and the absorbance of each sample was quantified by a spectrophotometer with absorbance at 530 nm.

### Amylase and Cellulase Production

To determine amylase and cellulase production, yeast nitrogen base (YNB) agar medium containing 6.7 g/l YNB, 2.0 g/l soluble starch, 0.5 g/l cellobiose, 1 g/l carboxymethyl cellulose, and 20 g/l of agar was used. The reactants were solubilized in warm water, the pH adjusted to 7.0, autoclaved for 15 min at 120°C and then poured into 150 mm × 20 mm Petri dishes. Using a multi-inoculator, the isolates from the matrix plate were transferred and grown in this medium for 24 h at 28°C. To reveal the production of amylases, the plate was covered with a lugol solution for 5 min. This solution was then discarded and the production of a transparent halo in the culture medium was evaluated (Strauss et al., 2001). To evaluate cellulase production, a solution of 0.03% Congo Red was added, for 10 min, to the culture medium, followed by washing with a 1 M NaCl solution. Cellulase producing bacteria formed a clear halo on a red background (Strauss et al., 2001).

### Siderophore Production

The detection of siderophore production was based on the Schwyn and Neilands method (Schwyn and Neilands, 1987). From the matrix plates, 3 μl of a solution of isolates (0.6–0.8 OD_600nm_) was transferred to 96-well plates containing 200 μl of CN medium, previously treated with 3% (w/w) 8-hydroxyquinoline. After 2 days at 28°C, the change in color from blue to yellow around the colony indicated the production of siderophores.

### Phosphate Solubilization

For phosphate solubilization assays, the NBRIP (National Botanical Research Institute’s Phosphate) culture medium was used (Nautiyal, 1999). The isolates were originally grown on a matrix plate containing CN medium, and then ∼3 μl of a solution of isolates (0.6–0.8 OD_600nm_) was transferred to 150 mm × 20 mm Petri dishes containing NBRIP culture medium. After 3 days at 28°C, the evaluation of the formation of a clear halo in the medium indicated the ability of phosphate solubilization by the isolate.

### Evaluation of Antibiotic Tolerance

From the matrix plates, 3 μl of a solution of isolates (0.6–0.8 OD_600__nm_) was transferred to 96-well plates containing 200 μl of CN liquid medium and either 100 μg/ml ampicillin or 30 μg/ml tetracycline. After 3 days at 28°C, the ability of the bacterial strains to grow in the respective antibiotics was evaluated.

### Arsenic Tolerance

From the matrix plates, 3 μl of a solution of isolates (0.6–0.8 OD_600__nm_) was transferred to 150 mm × 20 mm Petri dishes containing LB agar with sodium arsenite (NaAsO_2_) at 1, 5, and 10 mM concentrations. After 3 days at 28°C, the ability of the bacterial strains to grow in the respective arsenic concentrations was evaluated.

### Morphological Characterization

The isolates were grown in LB medium at 28 ± 2°C and 150 rpm in a shaker for 24 h and then Gram stained. The isolates were observed under an optical microscope with 100× magnification and were then characterized based on their morphology and coloration.

### Growth Curves

The bacterial isolates were grown in CN medium for 12 h at 28 ± 2°C and at a rotation of 150 rpm. The bacterial density was standardized for all isolates at OD_600__nm_ = 1.0 (∼10^8^ cells/ml). The cell suspension was then diluted to 1:500 in CN medium and the optical density monitored over time at 600 nm.

### Extraction of Plasmid DNA

An isolated colony was inoculated into CN medium and incubated at 28 ± 2°C for 12 h. Plasmid DNA extraction was performed using the QIAprep Spin Miniprep Kit^TM^ extraction kit. For visualization of the extracted plasmid DNA, the samples were submitted to 0.7% agarose gel electrophoresis.

### Bacterial Transformation by Electroporation

*Escherichia coli* strain Stbl2 [F-endA1 glnV44 thi1 recA1 gyrA96 relA1 Δ (lac-proAB) mcrAΔ (mcrBC-hsdRMSmrr) λ-] was inoculated into CN medium at 28 ± 2°C with a rotation of 150 rpm. One hundred milliliter of CN medium was inoculated with 1 ml of the culture, grown for 12 h and the optical density monitored at 600 nm, to ∼ 0.5. Cells were centrifuged at 1.957 ×*g* for 5 min at 4°C. Thereafter, the cell pellet was resuspended in 50 ml of cold sterile distilled water and centrifuged twice, as before. The cells were resuspended in 10 m of cold sterile 10% glycerol, incubated on ice for 5 min and centrifuged as above, followed by the addition of 300 μl of 10% sterile glycerol. Forty microliter of the cell suspension was used for each transformation reaction. Plasmid DNA was added to the cell suspension and incubated on ice for 5 min. The mixture was transferred to an electroporation cuvette and subjected to a 1.8 kV pulse. Immediately, 1 ml of SOC medium (2 g/l tryptone, 0.5 g/l yeast extract, 0.05 g/l NaCl, 0.02 g/l KCl, 0.8 g/l glucose, and 40 mM MgCl_2_) was added to the cell suspension and incubated at 28 ± 2°C overnight. One hundred microliter of cell suspension from each reaction was plated onto LB solid medium containing the appropriate antibiotics.

### Plasmid Stability Assay

The plasmid stability assay was performed using the method of Yao et al. (2015). Plasmid-containing *E. coli* Stbl2 cells were inoculated into LB medium supplemented with 100 μg/ml ampicillin and incubated at 28 ± 2°C and at a rotation of 150 rpm for 24 h. Bacterial suspension aliquots were withdrawn and normalized to optical density equal to 1.0 (∼10^8^ cells/ml), followed by serial dilutions up to 10^-5^. One hundred microliter of the last dilution was plated in LB solid medium, some of which were supplemented with 100 μg/ml ampicillin, and after 24 h of growth the colonies were counted. Procedures were repeated for 6 consecutive days.

### Colony Forming Units Count

Cells were grown in liquid LB medium for 12 h at 28 ± 2°C and shaking at 150 rpm. Bacterial cell density was standardized for all isolates in OD equal to 1 (10^8^ cells/ml). The cell suspension was diluted to 1:500 in LB medium and incubated at 28°C and 150 rpm with 1, 2, and 5 mM of sodium arsenite and the optical density was monitored until reaching a value equal or close to 1. The bacterial suspension was diluted to ∼10^-6^ cells/ml, followed by plating of 100 μl in solid LB. The plates were incubated at 28 ± 2°C for 24 h and the number of forming units was evaluated.

### Plant Growth Test

Twenty Santa Clara 5800 tomato seeds were sown in soil prepared with compound perlite and vermiculite (6:4) and stored at 4°C for 3 days in the absence of light, to accelerate germination. After this period, 10 ml of water containing the isolate, in optical density equal to 1.0 (∼10^8^ cells/ml), was inoculated into the soil. The tomato seeds were planted in soil and transferred to the greenhouse of The Federal University of Ouro Preto for 21 days under controlled conditions (26°C and 60% humidity). Then, the germination rate was evaluated and the aerial parts of the plants were measured.

### Autoaggregation Assay

This assay was based on the protocol of Alamuri et al. (2010). Cultures of bacterial isolates, grown overnight in CN medium at 28 ± 2°C, were adjusted to the same optical density (OD = 1.0), and 10 ml samples from each culture were transferred to 20 ml sterile tubes. Initially, all cultures were shaken vigorously for 15 s. The tubes remained static throughout the experiment and 100 μl samples from each tube were taken from ∼ 1 cm from the top of the culture and the optical density was evaluated at OD_600__nm_ every hour.

### Biofilm Production Assay

This assay was based on the O’Toole protocol (O’Toole, 2011). Bacterial isolates were grown in LB liquid medium overnight at 28 ± 2°C. Bacterial density was standardized for all isolates in optical density equal to 1.0 (∼10^8^ cells/ml). The samples were then diluted to 1:10 in LB medium and 100 μl was transferred to a 96-well plate and incubated for 12 h at 28°C. After 12 h growth, the plate was washed with distilled water to remove the cells and allowed to dry for 2 h. Then, 125 μl of 0.1% crystal violet solution (CV) was transferred into each well and allowed to stand for 45 min. After incubation the plate was washed again with distilled water and allowed to dry. Then, 125 μl of 95% ethanol was added to each well and left for 45 min for the complete dissolution of the CV. The absorbance was recorded on a plate reader (Perkin Elmer VICTOR X3, Waltham, MA, United States) with a wavelength of 550 nm. For each isolate, seven replicates were performed.

### Quantification of Total Arsenic by X-ray Fluorescence Spectroscopy

To evaluate whether the isolates were removing arsenic from the culture medium, the cells were grown in 50 ml XVM2 medium [NaCl 1.16 g/l, (NH_4_)_2_SO_4_ 1.32 g/l, 1 mM CaCl_2_, 5 mM MgSO_4_, 0.021 g/l KH_2_PO_4_, K_2_HPO_4_ 0.055 g/l, FeSO_4_ 0.0027 g/l, fructose 1.8 g/l, sucrose 3.432 g/l, casamino acid 0.003 g/l, pH 7.0], supplemented with 1 mM sodium arsenite for 7 days. Aliquots of 1 ml were collected and centrifuged for 15 min at 5 ×*g* and 500 μl of the supernatants were collected for arsenic dosing. For total arsenic dosage, Total Reflection X-ray Fluorescence methodology was used (S2 PicoFox, Bruker, United States). Ten microliters of the sample were placed into quartz plates and the reading was performed with 600 s reading time, current 700 mA and voltage 50 kV.

### Scanning Electron Microscopy and Energy Dispersive X-ray (EDX) Analysis

Cells were grown in 50 ml XVM2 medium for 72 h, some containing 1 mM of sodium arsenite. Cells were collected and washed with phosphate buffer pH 7.2 three times, with centrifugation at 1.957 ×*g* for 5 min. The cell pellet was resuspended in phosphate buffer supplemented with 1% paraformaldehyde and 2% glutaraldehyde then placed on slides and allowed to dry for 2 h. The slides were dehydrated in an ethanol series of 30, 50, 70, 80, 90, 95, and 100%. The cells were coated with gold for electrical conduction and visualized under the scanning electron microscope (SEM) (Q150RES, Quorum).

### Arsenite Oxidase Activity Assay

The assays were performed using (with adaptations) the protocol of Dey et al. (2016). Cells were grown in 50 ml XVM2 liquid medium containing different concentrations of sodium arsenite at 28 ± 2°C and shaking at 150 rpm. Over 7 days, 500 μl of bacterial cultures were collected and centrifuged at 5,000 ×*g* for 15 min. One hundred microliter of the supernatant was mixed with 100 μl of 0.1 M AgNO_3_ solution. Yellow precipitate formation indicated the formation of Ag_3_AsO_3_ [silver arsenite (As^3+^)], while brown precipitate formation indicated the formation of Ag_3_AsO_4_ [silver arsenate (As^5+^)].

### Dosage of Intracellular Reactive Species

The assays were performed using (with adaptations) the protocol of Kim et al. (2017). Cells were grown in 50 ml LB liquid medium to the optical density equal to 1.0 (∼10^8^ cells/ml). One milliliter of the bacterial culture was collected and centrifuged at 1.957 ×*g* for 5 min. The supernatant was discarded, and the cells were washed three times with PBS (pH 7.2) and centrifuged as previously described. The cell pellet was resuspended in 1 ml of PBS at a final concentration of 20 μM of the 2′, 7′-Dichlorodihydrofluorescein (H_2_DCFDA) diacetate probe. The samples were incubated for 1 h at 37°C in the absence of light. The cells were then washed with PBS three times and resuspended in the same buffer, with a final concentration of 5 mM sodium arsenite, for 1 h at 37°C. Subsequently, 150 μl of the bacterial suspension was placed into 96-well plates and fluorescence emission was evaluated in a plate reader (Perkin Elmer VICTOR X3, Waltham, MA, United States) with excitation at 485 nm and emission 535 nm.

### Catalase Activity

The assays were based on the method of Aebi (1984). Cells were cultured in 50 ml CN medium at 28 ± 2°C until reaching optical density of 1.0 (∼10^8^ cells/ml). Thereafter, the bacterial culture was exposed to 5 mM sodium arsenite for 1 h. Cells were collected and centrifuged at 1.957 ×*g* for 15 min, washed three times with PBS buffer (pH 7.2) and the cell pellet was resuspended in 3 ml of PBS. Glass beads were added to the cell suspension and the tube was vortexed seven times for 30 s, with 30 s intervals on ice. One milliliter of the prepared extract was collected and centrifuged at 5 ×*g* for 15 min. Twenty microliters of the supernatant was added to the PBS solution with a final concentration of 1 mM H_2_O_2_. The absorbance reading was performed in a spectrophotometer (Biospectro SP-220) at 240 nm for 3 min. The protein dosage was performed by the Bradford method (Bradford, 1976).

### DNA Integrity

Bacterial cultures were grown in 50 ml LB liquid culture medium at 28 ± 2°C and rotated at 150 rpm until reaching the optical density of 1.0 (∼10^8^ cells/ml). Thereafter, the cultures were exposed to a final concentration of 0.5, 1, 2, and 5 mM of sodium arsenite for 12 h. After the exposure time, an optical density was normalized to 1.0. A volume of 1.5 ml was collected and centrifuged at 13.22 ×*g* for 5 min. The supernatant was discarded and the cells were resuspended in 300 μl of a solution containing 50 mM glucose solution, 25 mM Tris, 10 mM EDTA, pH 8.0. Five microliter of proteinase K (20 mg/ml) and 30 μl of 10% SDS were added. The mixtures were incubated at 37°C for 2 h. Then, an equal volume of chloroform was added, mixed, and centrifuged at 13.22 ×*g* for 5 min. The supernatant was collected, an equal volume of chloroform was added and the centrifugation was repeated. The supernatant was then collected and 0.1 volume of 3 M NaCl and 1 volume of ice-cold isopropanol were added. The mixture was allowed to stand for 3 h in the freezer at -20°C, and then centrifuged for 15 min at 13.22 ×*g*. The supernatant was discarded again and the precipitate was washed with 70% ethanol. The precipitate was allowed to dry at room temperature and then resuspended in 100 μl of TE buffer (pH 8.0). Integrity of the genomic DNA was evaluated on 1% agarose gel compared to a condition where the isolates were not exposed to sodium arsenite.

### Motility Assay

The assays were based on the protocol of Farasin et al. (2017). Bacterial cultures were grown in CN medium at 28 ± 2°C with rotation of 150 rpm until reaching an optical density equal to 1.0 (∼10^8^ cells/ml). Then, 10 μl of the bacterial suspension was dropped in LB semisolid medium (0.3% agar) containing 0.2 mM sodium arsenite or sodium arsenate. After 24 h of incubation at 28 ± 2°C, the plates were visually inspected and the diameters of respective colonies were measured.

### PCR Assay

Each PCR mixture contained 50 ng of template DNA, 2.5 μl buffer reaction (10X), 1.5 μl dNTP (10 mM), 2.5 μl MgCl_2_ (25 mM), 0.25 μl *Taq* polymerase (5 units/μl), 8 μl of the mixture Forward (GCTTGGGCATAGGTTGGAGT) and Reverse (GGCTCGACGTTTTTACGCAG) primers (10 pmol/μl) and sufficient water to reach 25 μl. PCR cycles were preceded by denaturation at 94°C for 3 min, followed by 35 consecutive cycles with 45 s at 94°C for denaturation, 45 s at 60°C for annealing and 45 s at 72°C for extension, followed by a final step of extension at 72°C for 3 min using Biocycler thermocycler (Biosystems, United States).

### Molecular Identification of Strains

For molecular identification of FG3, C25, and FOB3 strains, the genomic DNA was extracted by the Wizard^®^ Genomic DNA Purification Kit (Promega). To identify the strains, an amplicon was generated by PCR technique for the V4–V5 region of the 16S ribosomal gene. A total volume of 30 μl of reaction was used: 50 ng template DNA, 2.5 μl reaction buffer (10X), 1.5 μl dNTP (10 mM), 2.5 μl MgCl 2 (25 mM), 0.25 μl Taq polymerase enzyme (5 units/μl), 4 μl of the primer mixture Forward (GTGCCAGCMGCCGCGGTAA) and Reverse (CCGTCAATTYYTTTRAGTTT) (10 pmol/μl). PCR cycles were preceded by denaturation at 94°C for 3 min, followed by 35 consecutive cycles with 45 s at 94°C for denaturation, 45 s at 57°C for annealing and 45 s at 72°C for extension, followed by a final extension step at 72°C for 3 min, using 2720 ThermalCycler^TM^ (Applied Biosystems, United States). The amplicons generated by PCR were verified in 1% agarose gels and purified using 20% PEG–8000 in 2.5 M NaCl (Arbeli and Fuentes, 2007). The product obtained was quantified by spectrophotometry using NanoDrop ND 1000^TM^ (NanoDrop Technologies). Sequencing was carried out with the DYEnamicTM^TM^ kit (Amersham Biosciences, United States) in combination with the MegaBACE 1000^TM^ automated sequencing system (Amersham Biosciences, United States). The sequencing reactions were performed with 100–150 ng purified DNA and the reagents in the DYEnamic^TM^ kit (Amersham Biosciences, United States), following the manufacturer’s recommendations. The program consisted of 36 cycles with an initial denaturation at 95°C for 25 min, followed by 15 s annealing at 50°C and 3 min of extension at 60°C. After cycling, the reaction product was transferred to a 96-well sequencing plate to be precipitated. For precipitation, the established protocol was the same as previously described by Felestrino et al. (2017b).

### Nucleotide Sequence Accession Numbers

The sequences of FG3, C25, and FOB3 were deposited in GenBank, and the identifiers of these sequences are, respectively, MH424466, MH42447, and MH42448.

### Statistical Analyses

Statistical analyses were performed using the statistical package GraphPad Prism, version 5.00 (San Diego, CA, United States). The results were submitted to the normality test of Smirnov Kolmogorov and represented as the mean ± SEM. The Student’s *t*-test was used to compare pairs of parametric groups while one-way analysis of variance (ANOVA) was used to compare three or more groups with Tukey *post hoc* tests for parametric data. The Kruskal–Wallis test was used to compare Dunn’s *post hoc* test data, considering ^∗^*p* < 0.05, ^∗∗^*p* < 0.01, and ^∗∗∗^*p* < 0.001.

## Results

### Characterization of Isolates

Two canga regions were selected for collecting plant and soil samples: Canga Mãe d’Água and Canga Jardim Canadá, located in Sinclinal Moeda, and Nova Lima, respectively. The choice was based on the fact that they are inserted in a region of extreme conservation for the flora biodiversity of the state of Minas Gerais (**Figure 1A**). From samples of nine plants, some of which were from ferruginous rupestrian grasslands, restricted from cangas and from six soil samples (**Figure 1B**), 65 isolates were obtained (**Table 1**).

**FIGURE 2:**
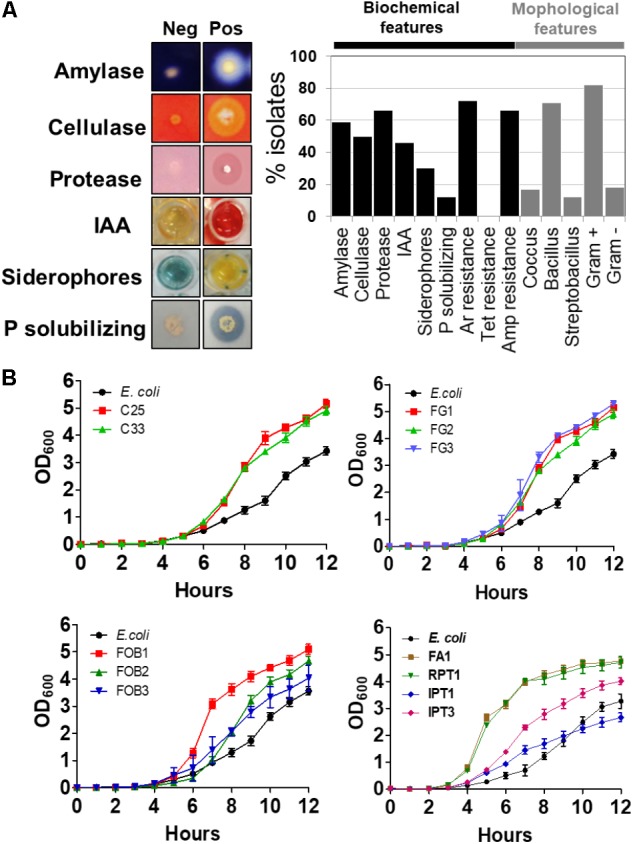
Biochemical and morphological characterization of the isolates using different assays. The results are expressed as the percentage of positive isolates for each assay **(A)**. Comparison of the growth curves of different plant and soil isolates to the control *Escherichia coli* **(B)**.

Once isolated and stored in a 96-well matrix plate, a series of qualitative biochemical assays and morphological characterization of these isolates were performed (**Figure 2A**). For these assays, 38 (58.46%) isolates were able to secrete amylases, 31 (47.69%) were able to secrete cellulases, 41 (63.07%) were able to secrete proteases, 30 (46.15%) were able to produce IAA, 17 (26.15%) were able to produce siderophores and only 6 (9.23%) of these isolates were able to solubilize inorganic phosphates (**Figure 2A**). None of the isolates were resistant to the antibiotic, tetracycline, although 44 (64.61%) isolates showed tolerance to ampicillin. Finally, 45 (69.61%) isolates were able to grow in culture medium containing 1 mM sodium arsenite (**Figure 2A**). Regarding morphology, 46 (71%) isolates were classified as bacilli, 10 (15%) as cocci and 9 (13.20%) as streptobacilli, and among these, 53 (81.13%) isolates were identified as Gram-positive and 12 (18.86%) as Gram-negative bacteria (**Figure 2A**). In order to verify the *in vitro* growth profile of these isolates, 11 were randomly selected. From the same cell concentration (∼10^8^ cells/ml), 10 of these isolates (C25, C33, FOB1, FOB2, FOB3, FG1, FG2, FG3, FA1, RPT1) showed a growth profile higher than *E. coli* when underwent to the same growth conditions. Only the IPT1 isolate showed a growth profile equal to or less than *E. coli* (**Figure 2B**).

### Evaluation of Potential as Biofertilizers

**FIGURE 3:**
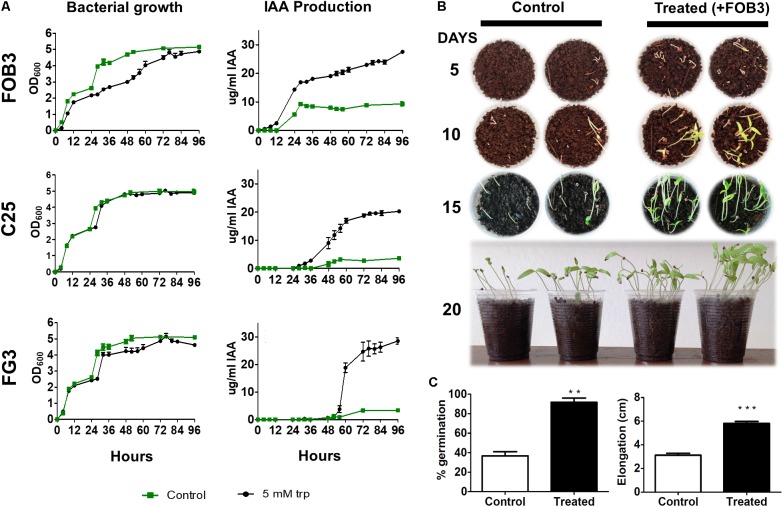
Evaluation of the potential of three bacterial isolates as PGPB. **(A)** Relationship between growth curve and IAA dosage in the presence and absence of tryptophan (Trp). The FOB3 isolate showed the highest rate of IAA production in the absence of Trp. **(B)** Tomato seeds were grown in the absence and presence of the FOB3 bioinoculatory potential and evaluated for 20 days. **(C)** The germination rate and growth of the aerial part were evaluated at the end of 20 days of incubation. The *p*-values were determined by Student’s *t*-test and the groups were considered significantly different when ^∗∗^*p* < 0.01 or ^∗∗∗^*p* < 0.001.

Among the 30 isolates that presented biofertilizer potential, for producing different concentrations of IAA, 3 were chosen because they presented the capacity to produce siderophores as well. Analysis of the 16S rRNA gene sequence corresponding to the V4–V5 region, showed that FG3, C25, and FOB3 correspond, respectively, to *Serratia* non-pigmented (ID MH424466), *Acinetobacter* (ID MH424467), and *Rosenbergiella* (ID MH424468). These bacterial strains were used for plant growth promotion trials. Initially, it was verified whether the production of IAA and the growth rate of these isolates were dependent on tryptophan and also if that the three isolates showed no significant variation in the growth rate, in the presence or absence of tryptophan (**Figure 3A**). At the same time, it was found that the production of IAA reached a peak of ∼ 30 μg/ml after 96 h with isolates C25 and FOB3, whereas the FG3 isolate produced ∼20 μg/ml of IAA in this same period, in the presence of tryptophan. However, in the absence of tryptophan, it was verified that only the FOB3 isolate produced significant amounts of IAA (10 μg/ml) within 20 h of culture, stabilizing its production up to 96 h of culture. On the other hand, the isolates C25 and FG3 produced only 2 μg/ml IAA throughout the whole culture period investigated (**Figure 2A**).

Based on these results, the FOB3 isolate was selected as the most promising in the plant growth promotion assay. For plant growth trials, 20 Santa Clara tomato seeds were sown in the presence and absence of the FOB3 isolate, according to methodology. The germination and elongation rates of aerial parts were verified over the course of 20 days. It was observed during all the gauging periods that the development of tomato plants in the presence of the FOB3 isolate was earlier and remained more efficient throughout the investigated period (**Figure 3B**). Plants inoculated with the FOB3 isolate showed a 100% germination rate and around 5.80 cm aerial part elongation compared to 36.7% germination and around 3.10 cm of aerial part elongation in plants not inoculated with this isolate (**Figure 3C**).

### Analysis of Plasmid Potential

**FIGURE 4:**
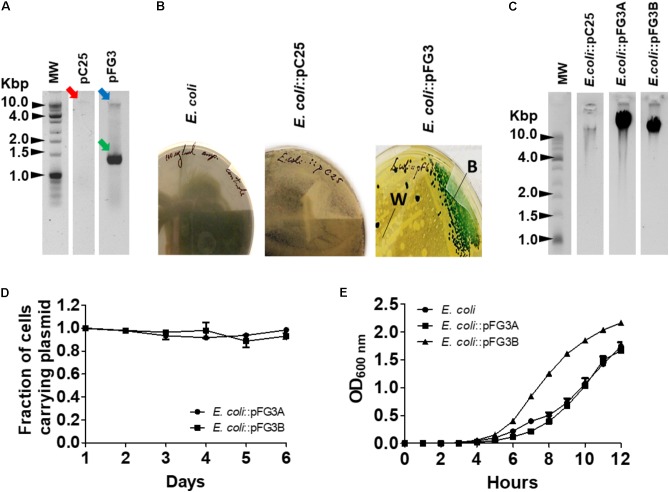
Characterization of the plasmids obtained from the isolates C25 and FG3. **(A)** Analysis of the electrophoretic profile of plasmids extracted from wild strains C25 (red arrow) and FG3 in two conditions, relaxed (blue arrow) and coiled (green arrow). **(B)** Phenotypic results of *E. coli* transformed with the C25 and FG3 plasmids and incubated in LB broth supplemented with X-gal and ampicillin. Two phenotypic profiles were observed for *E. coli*::pFG3, white (W) and blue (B) colonies suggesting the presence of two distinct plasmids in the wild strain. **(C)** Electrophoretic profile of the plasmids from the transformed *E. coli* strains. For C25 a plasmid of the same size as that of the wild strain (pC25) was observed. For FG3 two different plasmids, named FG3A (pFG3A) and FG3B (pFG3B), respectively, were observed. **(D)** Verification of the plasmid stability of transformed strains resistant to ampicillin and grown on LB agar and evaluated over 6 days of growth. **(E)** Growth curves of the transformed strains compared to that of wild-type *E. coli*, demonstrating that the plasmid pFG3B confers increased bacterial replication rate (as observed in **Figure 2B**).

Since plasmids are often related to both antibiotic and heavy metal tolerance, the possible presence of plasmids was verified for FOB3, C25, and FG3 isolates. Based on the established protocol, plasmids were identified in isolates C25 and FG3, but not in FOB3 (**Figure 4A**). In an attempt to understand the biological potentials associated with these plasmids, they were inserted into *E. coli* by electroporation. The transformed *E. coli* strains were incubated in a rich medium, containing ampicillin and X-gal, and after 24 h of growth it was found that the transformant carrying plasmid of the C25 isolate (*E. coli*::pC25) was resistant to ampicillin but unable to degrade X-gal (**Figure 4B**). The same procedure was performed for the FG3 plasmid, where the presence of white and blue colonies was observed, indicating the possibility of two plasmids. After isolation of these transformed colonies, two differentiated transformants were identified, respectively, named *E. coli*::pFG3A (β-gal+ and Amp+) and *E. coli*::pFG3B (β-gal- and Amp+) (**Figure 4B**). A further extraction of the plasmids from the transformed *E. coli* strains, confirmed that FG3 has two plasmids with small size variation (**Figure 4C**), confirming the qualitative results in the presence of X-gal.

To evaluate the stability of the plasmids in the transformed cells, *E. coli*::pFG3A and *E. coli*::pFG3B were cultured in an LB broth medium supplemented with ampicillin (100 μg/ml), and every 24 h a sample of these cultures was plated on solid LB medium containing antibiotic. It was observed that even after 6 days of culturing (∼450 replications) about 98 and 99% of the transformed cells (*E. coli*::pFG3A and *E. coli*::pFG3B, respectively), maintained the plasmids (**Figure 4D**). In an attempt to verify if the presence of the plasmids could interfere with the growth rate of *E. coli*, growth curves with the transformed strains were performed. It has been found that the presence of plasmid pFG3B in *E. coli* cells is capable of altering the growth rate, reaching the log phase of growth more rapidly compared to wild *E. coli*. In addition, *E. coli*::pFG3B showed a higher growth rate in relation to the wild-type FG3 isolate, a result not observed for the *E. coli*::pFG3A strain (**Figure 4E**).

**FIGURE 5:**
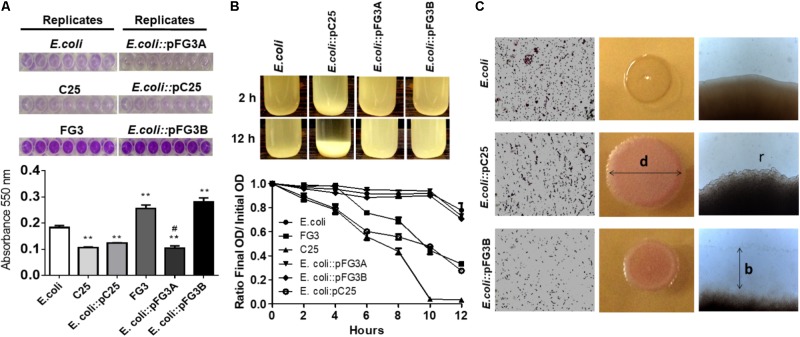
Verification of biofilm production and cell aggregation in transformed strains. **(A)** Analysis of the biofilm production in the wild-type and transformed strains incubated for 12 h and stained with crystal violet (seven replicates). The biofilm production was measured by spectrophotometry at 550 nm. The *p*-values were determined by one way ANOVA followed by Tukey’s *post hoc* test and the groups were considered significantly different when ^∗∗^*p* < 0.01 compared to wild *E. coli* and ^#^*p* < 0.05 compared to wild-type FG3. The high level biofilm production of the wild-type FG3 strain is mediated by the plasmid pFG3B. **(B)** Aggregation profile of the transformed strains after 2 and 12 h of rest in LB broth, starting from a culture with an OD_600_ of 1.0. To quantify this rate of aggregation, an approximate number of cells in the supernatant was estimated by spectrophotometry (OD_600_), measured every 2 h and expressed as mean ± SEM. *E. coli*::pC25 had an aggregation rate of ∼50% higher relative to wild-type *E. coli* or *E. coli* transformed with plasmids pFG3A and pFG3B, after 10 h. C25 and FG3 wild-types had an aggregation rate of 100 and 50%, respectively. **(C)** Optical microscopy showing aggregation profile altering diameter (d) colony roughness (r) in *E. coli*::pC25 strain and increase in biofilm production (b) around colonies of *E. coli*::pFG3B strain.

In order to characterize the potential of these plasmids, the *E. coli* transformed cells were evaluated for biofilm production and autoaggregation, in comparison to the wild strains C25, FG3, and *E. coli*. The wild C25 isolate and *E. coli*::pC25 transformant strain did not produce significant amounts of biofilm (**Figure 5A**). In contrast, it was observed that the FG3 isolate produced significantly higher amounts than the wild strains of C25 and *E. coli*. Interestingly, this effect was associated with the presence of the plasmid pFG3B, since the transformant *E. coli*::pFG3B showed levels similar to the wild-type FG3 isolate, an effect not observed for the *E. coli*::pFG3A strain (**Figure 5A**). As for the autoaggregation profile, the strains *E. coli*::pFG3A and *E. coli*::pFG3B were not able to form precipitates. However, *E. coli*::pC25 presented 73% of its aggregated cells and formed a precipitate after 12 h of rest (**Figure 5B**). This variation in biofilm production and autoaggregation potential was confirmed by microscopy, allowing the observation of a biofilm layer around the colony *E. coli*::pFG3B not observed in the other strains, and a greater roughness at the ends of the colony of *E. coli*::pC25, not observed in the other strains (**Figure 5C**).

### Analysis of Arsenic Tolerance Profile

**FIGURE 6:**
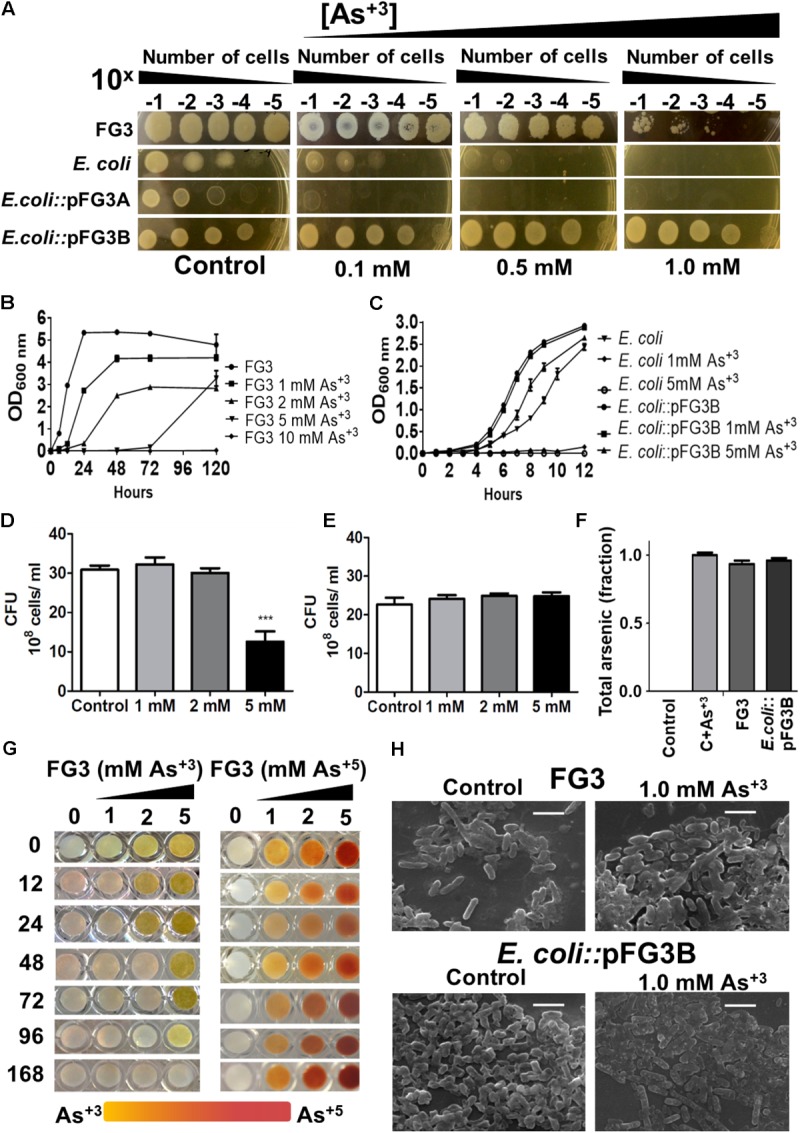
Evaluation of the tolerance, removal, and biotransformation of arsenic. **(A)** Relation between the number of cells and the tolerance to arsenic in culture medium, after 12 h of incubation at 28°C. **(B)** Growth curves of the wild-type FG3 isolate at different concentrations of sodium arsenite. **(C)** Growth curves of wild-type and transformed *E. coli* at different concentrations of sodium arsenite. It is possible to observe that the tolerance to arsenic is mediated by the presence of the plasmid pFG3B. **(D)** CFU of FG3 wild-type submitted in different arsenite concentrations, reiterating the results observed in **(B)**. **(E)** CFU of *E. coli*::pFG3b strain submitted in different arsenite concentrations, reiterating the results observed in **(C)**. **(F)** Analysis of Arsenic removal by wild-type and transformed strains. Data were calculated as ratio of initial to final arsenic concentration **(G)** Verification of the activity of the enzyme arsenite oxidase. FG3 strain was grown in XVM2 medium supplemented with different concentrations of arsenite and sodium arsenate. Over time, samples were collected and the supernatant was mixed with 0.1 M solution of Silver Nitrate in the ratio. **(H)** Strains were grown in XVM2 medium supplemented with 1 mM sodium arsenite for 7 days, dosing of total arsenic in the supernatant was performed by X-ray fluorescence spectroscopy and the cell morphology was examined by scanning electron microscopy. Results are expressed as the mean ± SEM (d) considering ^∗∗∗^*p* < 0.001. 1:1.

Due to high soil metal rates where the isolates were collected, all 65 isolates were challenged for arsenic tolerance. FG3 was one of the most tolerant bacterial strain to the presence of this metalloid (5 mM). In an attempt to understand whether this adaptation was plasmid-dependent, *E. coli*::pFG3A- and *E. coli*::pFG3B-transformed strains were incubated in culture media containing different concentrations of sodium arsenite. It was found that wild *E. coli*, as well as *E. coli*::pFG3A, did not show tolerance to high concentrations of arsenite. In contrast to these results, the *E. coli*::pFG3B-transformed strain presented a tolerance profile similar to the wild strain FG3 (**Figure 6A**).

To better evaluate this tolerance to the metalloid, growth curves at different concentrations of arsenite were performed. The FG3 isolate was shown to be able to grow at concentrations of 1, 2, and 5 mM. It is also noteworthy that at 5 mM, the strain had a longer stationary phase than to the other lower doses tested and then a very quick growth rate compared to the other cultures (**Figure 6B**). The *E. coli*::pFG3B strain was shown to be even more resistant when comparing its growth profile at the same arsenite concentrations to that of the FG3 isolate (**Figure 6C**). Note that the *E. coli*::pFG3B strain showed an almost identical growth profile when exposed to 1 mM of arsenite, while wild *E. coli* was not able to grow at any concentration of arsenite tested (**Figure 6C**).

To verify the viability of the cells, they were exposed to different concentrations of sodium arsenite, and when they reached the approximate concentration of 10^8^ cells/ml, the suspensions were diluted, plated and the colony forming units count was evaluated. It was found that FG3 has the same proportion of viable cells compared to the control at concentrations of 1 and 2 mM of arsenitebut, at the concentration of 5 mM this ratio reduces to approximately half of viable cells (**Figure 6D**). However, it was observed that at all concentrations of arsenite tested, the proportion of viable cells remained constant for *E. coli*::pFG3B (**Figure 6E**). We do not reject the hypothesis that complexes can be formed among the metals, phosphates, and other components present in the growth medium.

Aiming to better understand the relationship between tolerance and the ability to remove arsenic in the medium, wild-type FG3 and *E. coli*::pFG3B strains were cultured in XVM2 medium supplemented with 1 mM arsenite. The supernatants were collected and submitted to total arsenic dosing by the X-ray Fluorescence Spectroscopy method (**Figure 6F**). A culture medium containing 1 mM of arsenite was used as a positive control, and a culture medium without arsenite was used as a negative control. At the end of 7 days of growth, the percentage of removal of the metalloid in solution was quantified, where a low removal efficiency was observed for FG3 (5.78%) and *E. coli*::pFG3B (2.82%).

Since the strains are resistant but do not bioaccumulate arsenic, they may possibly act as biodegraders of the metalloid. To verify this hypothesis, cultures of FG3 cells were grown in XVM2 media containing different concentrations of arsenite and arsenate. Formation of a yellow color precipitate in the presence of silver nitrate indicates formation of silver arsenite (Ag_3_AsO_3_), while the formation of a brown precipitate indicates the presence of silver arsenate (Ag_3_AsO_4_). **Figure 6G** shows that with 12 h of experiment there was no formation of silver arsenite at a concentration of 1 mM of arsenite, demonstrating that this species had biotransformed it. At 48 h this result was repeated for a 2 mM concentration of arsenite, and from 96 h again this was repeated at a concentration of 5 mM. With 168 h of cultivation there was a complete disappearance of the precipitate of silver arsenite. Contrasting this arsenite biotransformation, no modification was observed when FG3 was grown in the presence of arsenate under the same conditions.

To investigate whether the presence of this metalloid induces a change in cell morphology, strains FG3 and *E. coli*::pFG3B were grown in culture media containing 1 mM arsenite, and their morphology was evaluated by SEM. As can be seen in **Figure 6H**, no apparent change in the morphology of FG3 or *E. coli*::pFG3B was observed. Similarly, analysis by energy-dispersive X-ray (EDX) was not able to detect the presence of arsenic in the cell composition of either strain. These results corroborate that both FG3 and *E. coli*::pFG3B do not bioaccumulate or adsorb arsenic in their membranes.

### Induction of Redox Processes

**FIGURE 7:**
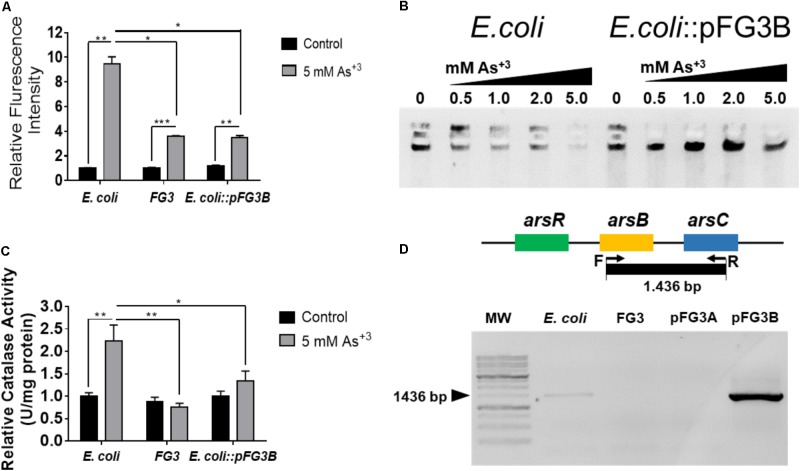
Verification of the induction of redox processes by sodium arsenite. **(A)** Production of reactive oxygen species (ROS) **(B)** catalase activity. It is possible to observe that the profile of catalase activity is proportional to ROS production in the strains investigated. The results were expressed as the mean ± SEM considering ^∗^*p* < 0.05, ^∗∗^*p* < 0.01, and ^∗∗∗^*p* < 0.001 by one way ANOVA followed by the Tukey’s *post hoc* test. **(C)** Evaluation of the integrity of genomic DNA from wild-type *E. coli* and *E. coli*::pFG3B in agarose gel after the treatment with increasing concentrations of arsenic. Note that the DNAs of the transformed strain maintained greater integrity than those of the wild-type strain at all concentrations investigated. **(D)** Amplification of genomic region of the arsB-arsC amplicon by PCR demonstrating that these genes are present in plasmid pFG3B.

To verify if the exposure to these metals induces redox processes in these strains, dosage of RS, consumption of peroxide and DNA integrity were investigated. As can be observed in **Figure 7A**, the production of reactive oxygen species (ROS) occurred in all tested strains when exposed to arsenic, however this increase was more prominent (∼2.5 times) in *E. coli* than in the strains FG3 and *E. coli*::pFG3B (**Figure 7A**). This data was corroborated by the evaluation of catalase activity under the same conditions established for the H_2_DCFDA experiments, indicating that in *E. coli* the induction of redox processes is higher than in the FG3 strain and in transformed *E. coli* (**Figure 7B**).

To verify if intracellular ROS were causing damage to DNA, its integrity was investigated in *E. coli* and in the *E. coli*::pFG3B-transformed strain when underwent to different concentrations of sodium arsenite. As shown in **Figure 7C**, *E. coli* exposure at different concentrations progressively increased the degradation of genomic DNA. On the other hand, it was observed that in the *E. coli*::pFG3B strain, the DNA remains intact even at the highest concentrations of arsenite.

In order to verify whether the arsenic tolerance conferred by plasmid pFG3B is related to the presence of *ars* genes, wild *E. coli* and FG3 strains and the plasmids of the transformed strains *E. coli*:: pFG3A and *E. coli*:: pFG3B were investigated. As it can be observed in **Figure 7D**, an amplification product corresponding to the theoretical amplicon of 1,436 bp was detected in *E. coli* genomic DNA and in plasmid pFG3B. However, no amplification product was observed for the FG3 genomic DNA and the plasmid pFG3A, indicating that the expression of the *ars* genes is involved in the tolerance of the strains to arsenic.

### Motility in the Presence of Arsenic

**FIGURE 8:**
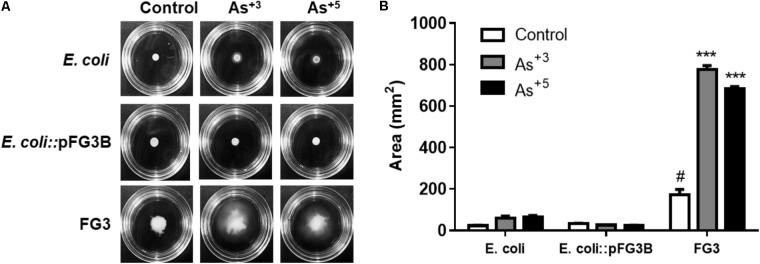
Analysis of arsenic-induced cell motility. **(A)** Swimming motility induced by arsenic in the wild-type FG3 strain, demonstrating that this protection mechanism is not associated with the presence of the plasmid pFG3B. **(B)** Quantification of the motility profile in the strains investigated. ^∗∗∗^*p* < 0.001 was considered significant in the groups compared to *E. coli* and ^#^*p* < 0.05 when compared to wild-type FG3 by one way ANOVA followed by Tukey’s *post hoc* test.

Another strategy for surviving stress is cell motility. *E. coli* and strains resistant to arsenic were evaluated for their swimming-type motility in semisolid media containing 0.2 mM of sodium arsenite or sodium arsenate. Motility was evaluated by the growth area of the colony observed in the culture medium. It was found that both *E. coli* and *E. coli*::pFG3B exposed to arsenite or arsenate did not exhibit swimming-like motility (**Figure 8A**). However, it was observed that, under normal conditions, FG3 presented a larger area of growth compared to *E. coli*, and when exposed to arsenite and arsenate, increased its motility compared to the respective control (**Figure 8B**).

## Discussion

The cangas are outcrops formed millions of years ago as a result of the weathering of iron-bearing rocks that are structured in armor that can reach 10s of meters of thickness and extend over 1000s of hectares (Carmo and Jacobi, 2013). In the IQ region, these areas are colonized by plants that present adaptations to limiting conditions such as shallow soils, water deficit, low fertility, high daily thermal amplitudes, high incidence of fire, sun exposure and constant winds (Jacobi et al., 2007). All plants growing on ferruginous outcrops are metallophytes, or specifically pseudo-metalophiles, as many of them are capable of bioaccumulating metals as an anti-herbivory feature (Ribeiro et al., 2017). For these reasons, and given their ecological importance in extreme environments, plants of this environment have been well-studied (Silveira et al., 2016). However, studies that portray the importance of the microbiota associated with these plants are still incipient, and its biotechnological potential is practically unknown.

Recent works developed by our team have demonstrated that the microbial composition associated with these plants is an important factor for such adaptations (Felestrino et al., 2017a,b). Continuing these investigations, this work aimed to understand the biotechnological potential of the cultivable microbiota associated with plants of the ferruginous rupestrian grasslands. For this, 65 bacteria were isolated from plants and plant soils in the state of Minas Gerais.

Initially, the ability of the isolates of this study to act as PGPB was evaluated, and a series of qualitative exploratory tests were proposed. Regarding the production of hydrolytic enzymes, ∼58% of the isolates were amylase producers, 63% cellulases and 47.69% proteases. The secretion of these enzymes in the environment can play a fundamental role in plant growth by promoting the cycling of organic matter in the soil (Choubane et al., 2016). In the case of canga soils, which are reported to have low fertility, these isolates may be of crucial importance in the cycling of essential nutrients for the maintenance of the plants. In addition, they can indirectly act as biocontrollers for other organisms, thus protecting against damage to cellular structures (Glick, 2012). In view of these findings, future research on the potential of these enzymes opens a new perspective of investment related to the microbiota associated with ferruginous rocky fields.

Similarly, the siderophores can act as biocontrollers, produced by about 25% of the isolates. They are low molecular weight molecules that have high affinities for iron and other metals (Cabaj and Kosakowska, 2009; Mahanty et al., 2017) and these molecules can limit the availability of metals to other organisms and plants, also acting as indirect promoters of plant growth (Ahmed and Holmstrom, 2014; Mahanty et al., 2017).

Also investigated in this work were the abilities to solubilize phosphate and to produce IAA by culturable isolates, characteristics desirable for PGPB. A small proportion of the isolates evaluated (9.23%) were able to solubilize inorganic phosphate. However, canga regions have very low concentrations of phosphorus, usually found in the form of phosphates (Vincent and Meguro, 2008; Schaefer et al., 2015). Thus, although the number of isolates with this capacity is limited, those that have it might have fundamental importance for the maintenance and establishment of plants in these regions. Regarding the ability of bacteria to produce IAA, 46.15% of the isolates showed such potential, reaching concentrations ranging from 2 to 10 μg/ml in the absence of tryptophan. The outcrops on the cangas are very hard, which can be an obstacle for deepening the roots of plants (Skirycz et al., 2014), the production of this phytormonium by almost half of the isolates should contribute to the establishment of these plants in such an environment, helping to promote the branching of their roots into the soil in search of nutrients. The set of isolates capable of solubilizing phosphate and producing IAA may be biotechnologically investigated for potential use as agricultural bioinoculants in soils lacking in nutrients, being, therefore, able to establish planting or increase productivity (Adnan et al., 2017).

In an attempt to empirically understand the importance of these bacteria in promoting plant growth, three isolates (C25, FOB3, and FG3), which were shown to be most promising as PGPB, were evaluated for their ability to promote growth in tomato plants. Firstly, the possibility of producing IAA in the absence of the amino acid L-tryptophan, a key precursor for phytonutrient synthesis, was evaluated, as the use of tryptophan-dependent PGPB for IAA synthesis becomes economically unviable for application as bioinoculants in large scale farming or restoration of degraded areas. After 98 h of culture in the presence and absence of tryptophan, it was possible to verify that the FOB3 isolate produced the highest amount of IAA in the absence of the precursor, ∼10 mg/l after 24 h of growth. When inoculated into soil containing tomato seeds, this isolate was able to raise the germination rate from 40 to 100% and increase the aerial part growth by 50%, from 3 to 6 cm after 20 days of growth (**Figure 3**). A study carried out by Khalid et al. (2004), showed that in the absence and presence of L-tryptophan, PGPBs that were able to produce between 12 and 24.8 mg/l IAA strongly stimulated the growth of wheat plants. Considering that FOB3 in the absence of the precursor was able to produce almost double the concentration of this same phytormonium, and that the result was promising in tomato plants, it is possible that it could also promote growth of other plants of agronomic interest, with the possibility of it being used as a tryptophan-free bioinoculant and would, therefore, have lower implementation costs.

Other outstanding characteristics of the isolates investigated relate to their ability to grow in antibiotic-containing culture media (ampicillin and arsenic at different concentrations). It is well-described in the literature that several plasmids are related to antibiotic and heavy metal tolerance, including arsenic tolerance. Among the three isolates investigated for the production of IAA, only in FOB3 was not observed the presence of an associated plasmid. In C25, one plasmid was obtained (pC25) while two plasmids (pFG3A and pFG3B) from FG3 isolate were obtained, all conferring tolerance to ampicillin in transformed *E. coli* strains.

Plasmid stability data showed that these plasmids have high stability within transformed cells, maintained through generations, which raises the potential of these plasmids as biological vectors. Another peculiar characteristic observed was the ability of the plasmid pFG3B to stimulate the growth of the transformed strain, raising its replication rate significantly, suggesting the existence of genes that directly or indirectly act in the control of the cell cycle. Further identification of such genes may allow new transformation and cloning vectors to be constructed in order to accelerate the production of recombinant enzymes of human interest, for example.

In addition, recent research describes high concentrations of arsenic in the IQ (Costa et al., 2015), mainly located in the center-north region. Chemically, the pentavalent form (arsenate) is more abundant in oxidizing environments, and the trivalent form in reducing environments (Roy and Saha, 2002; Watanabe and Hirano, 2013). It has been reported by Ghosh that one of the possible mechanisms of resistance to arsenate, a more abundant form in the surfaces, coincides with the non-solubilization of phosphates, since these are internalized in the cells through the same carriers, thereby preventing damage to biomolecules (Ghosh et al., 2015). Morphologically, the isolates from this study were classified as gram-positive bacilli. A similar study evaluated the resistance of two *Bacillus* gram-positive bacterial isolates to arsenic (Dey et al., 2016). According to the authors, the thicker wall of gram-positive bacteria may hinder the entry of toxic compounds, such as arsenic, which is consistent with the results found in this work, suggesting that the isolates from the canga regions are highly adapted to the conditions in which they live.

To survive, the bacteria must adapt quickly to changes in environmental conditions. Changes in growth rate must be accompanied by changes in the cell cycle to ensure that cell division is coordinated with mass doubling, chromosomal replication, and chromosomal segregation (Wang and Levin, 2009). It was possible to observe that a large number of the isolates tested had a high replication rate when compared to *E. coli* under normal conditions. We speculate that the bacteria of the canga regions, due to being in constant contact with an environment of high concentrations of toxic metals and few nutrients, need to multiply rapidly as part of a survival mechanism to guarantee their presence and perpetuation in the environment, even under unfavorable growth conditions.

For plant-associated bacteria, biofilm formation is an adaptive strategy to successfully achieve colonization of the host, for example, on leaf or rhizosphere surfaces, or even as a key strategy for pathogenesis, as well as protecting against environmental conditions (Castiblanco and Sundin, 2016). Our results show that, of the strains evaluated (**Figure 4A**), only the FG3 strain as the transforming *E. coli*::pFG3B strain produced significant amounts of biofilm, suggesting that the presence of the plasmid pFG3B directly influences the metabolism of cellular biofilm production. The data so far point to the plasmid pFG3B as a highly specialized molecule in the medium, as part of a mechanism essential to the adaptation of the strain to the canga regions. In addition, it was observed that the strain carrying the plasmid pC25 has a greater ability to autoaggregate compared to the others. Unfavorable growth conditions, or even low metabolic activity, are able to induce cellular aggregation, reflecting a strategy of survival in hostile environments (Bogino et al., 2013). Under favorable growth conditions, pC25 induces a greater capacity for cellular autoaggregation. According to Bogino, the ability of cells to autoaggregate has implications for agriculture in the production of bacteria-based inoculants. Bacterial aggregates can be produced on a large scale and separated in a simpler and easier way, in relation to the dispersive bacteria in the medium (Bogino et al., 2013).

Our data shows that both the FG3 strain and *E. coli*::pFG3B have high tolerance to sodium arsenite, growing even at high concentrations (**Figures 6A–E**). Arsenic removal analysis revealed that resistant strains do not bioaccumulate arsenic within cells or even in the cell membrane, with no apparent structural modifications. In addition, the ability of the FG3 strain to decrease the toxicity of the medium by oxidizing arsenite to arsenate, which was not observed in our experiments, was evaluated. This result may be related to the production of siderophores by the bacterium. According to Ghosh, bacteria producing a high number of siderophores are more resistant to arsenite, while bacteria producing a low number of siderophores are more resistant to arsenate (Ghosh et al., 2015). Our data suggests that siderophores produced by FG3 chelate with arsenite (As^3+^) in culture media as a way to attenuate the stress caused by this metalloid. The mechanism of resistance most widespread in bacteria is related to the functional presence of the operon *ars*, which can be associated with both chromosomal and plasmid DNA (Kaur and Rosen, 1992; Bachate et al., 2009). The presence of *ars* operon genes was found in plasmid pFG3B, not found in the genome of the FG3 strain, suggesting once again that this plasmid in particular has an important role in the adaptation of this strain to environmental adversity. The presence of arsenic tolerance genes and possibly other mechanisms not evaluated here, such as the production of siderophores and DNA damage response mechanisms, may be acting together to decrease intracellular toxicity and protect cells globally against damage to biomolecules, thus allowing greater resistance and adaptation to the adverse environment. These results also show that the plasmid pFG3B has prominent characteristics that might be used for biotechnological purposes.

Once bacteria detect toxic compounds around them, they can protect themselves by forming biofilms or by moving to less toxic areas (Farasin et al., 2017). It has been reported in literature that the proteome of the strain *Herminiimonas arsenicoxydans*, combined with transcriptome analysis results, indicated that *H. arsenicoxydans* not only expressed genes for detoxification and stress response against arsenic, but also genes involved in the synthesis of exopolysaccharides, phosphate, and motility (Weiss et al., 2009). It was observed that the FG3 strain is highly sensitive, presenting greater motility when exposed to arsenite and arsenate. This is clearly a mechanism of defense for this bacterium, against the toxicity of arsenic, suggesting that this mechanism is not related to the plasmid pFG3B.

## Conclusion

Our data demonstrate that the bacterial isolates from the canga regions of the IQ have ecological and biotechnological implications, which may contribute as PGPB in nutrient-poor soils, increasing the growth of plants, or even cycling organic compounds present in the environment through lytic enzymes. Our results also revealed that the plasmid pFG3B has desired characteristics for the biotechnology industry, such as metal tolerance and antibiotic resistance, and accelerating the growth of the host cells. A compilation of these characteristics (high stability, stimulation of the growth rate) for the creation of an expression vector could optimize and accelerate the production of enzymes, demonstrating its high biotechnological potential. The isolates from canga regions have an arsenal of genes with promising biotechnological potential, suggesting the need for further studies aimed at identifying new species, as well as characterizing of new interesting genes.

## Ethics Statement

The authorization for the collection of plant and soil samples was granted by the Instituto Chico Mendes de Conservação da Biodiversidade, issued by the number 54015-3, valid until 03/01/2019.

## Author Contributions

WC, ÉF, LM, FdC, and LK: designed the work, selected the plant samples investigated and collected the plant samples. WC, ÉF, IV, MV, IC, RA, CL, NF, and AS: performed all biochemical assays. WC, ÉF, LM, FdC, LK, and CG: interpreted findings. WC, LM, FdC, LK, AS, and CG: wrote the paper. WC, IV, IC, RA, CL, NF, AS, MV, FdC, LK, CG, and LM: contributed additional interpretations and general manuscript comments. WC, LM, RA, AS, FdC, LK, and CG: revised the paper.

## Conflict of Interest Statement

The authors declare that the research was conducted in the absence of any commercial or financial relationships that could be construed as a potential conflict of interest.
